# Periodic Optimization of Bus Dispatching Times and Vehicle Schedules Considering the COVID-19 Capacity Limits: A Dutch Case Study

**DOI:** 10.1177/03611981221114119

**Published:** 2022-08-25

**Authors:** Konstantinos Gkiotsalitis, Tao Liu

**Affiliations:** 1 Department of Civil Engineering, University of Twente, Enschede, Netherlands; 2 National Engineering Laboratory of Integrated Transportation Big Data Application Technology, School of Transportation and Logistics, Southwest Jiaotong University, Chengdu, China

**Keywords:** public transportation, bus transit systems, optimization, schedule, capacity and quality of service, capacity

## Abstract

The COVID-19 pandemic has had serious adverse impacts on public transport service providers. Most public transport lines exhibit reduced ridership levels while, at the same time, some of them may exhibit passenger demand levels beyond the pandemic-imposed capacity limitations. This study models the problem of bus dispatching time optimization within a periodic rolling horizon optimization framework that reacts to travel time and passenger demand variations. This model allows public transport service providers to adjust their bus schedules periodically to avoid in-vehicle crowding beyond the pandemic-imposed capacity limit. The proposed model is a mixed-integer linear program that considers the possible changes to vehicle schedules and tries to minimize the number of vehicles required to perform the service while adhering to the COVID-19 capacity restrictions. Case study results from the implementation of our model on bus Line 2 in the Twente region in the Netherlands are provided demonstrating the potential gains when rescheduling the trip dispatching times and vehicle schedules.

The COVID-19 pandemic has significantly affected the transportation sector, especially public transport (*1 [Bibr bibr2-03611981221114119]*–*3*). For example, at the initial outbreak stage, public transport systems in some cities, such as Wuhan and San Francisco, were completely shut down to slow the spread of COVID-19. In addition, public transport systems in major cities around the world have experienced a sharp decline in ridership. It is estimated that public transport ridership has declined by as much as 80% to 90% in major cities in the U.S. and China (*4*). A recent study shows that during the initial lockdown stage rail passenger ridership in the UK fell to about 5% of its normal level (*5*). The decline of passenger numbers has led to a reduction in public transport revenues, which has increased the financial burden on public transport agencies.

To maintain their service variability, public transport agencies have adopted various measures, such as social distancing, temperature screening, wearing face masks, hygiene, sanitization, and ventilation. Many public transport agencies have advised passengers to keep a physical distance of 1 to 2 m between themselves and other passengers to reduce the spread of the COVID-19 virus (*2*, *3*). The use of social distancing, however, significantly reduces the capacity of public transport vehicles. For example, a recent study showed that keeping 1.5 m social distancing on the Washington DC metro network would reduce carrying capacity by 80% (*6*). Another recent study further showed that the average seated train occupancy of the Washington DC metro would drop to between 19% and 28% for all its lines when implementing a 2 m social distancing policy (*7*). The implementation of different social distancing policies will lead to different usage capacities in public transport vehicles. Therefore, determining how to meet the social distancing requirements by optimally dispatching public transport vehicles is an interesting and timely topic. In addition, it will be valuable to further incorporate the uncertainties of vehicle running times and passenger demand in dispatching time optimization so as to provide more resilient and robust public transport services. In the particular case where the number of planned trips is not sufficient to meet the social distancing requirements, new trips can be added to the daily plan and their dispatching times and vehicle schedules can be updated. This study focuses on this direction by developing a mixed-integer linear program for the optimal rescheduling of the trip dispatching times and vehicle schedules to meet the social distancing requirements.

## Literature Review

The bus dispatching time optimization problem is part of the wider bus frequency setting and timetabling problem and it can be divided into two categories: single-line dispatching time optimization and multiple-line dispatching time optimization. Below we provide a concise review of the two categories.

Scheduling the dispatching times of several trips operating on a bus line is a multivariable optimization problem where the dispatching time of every trip is a decision variable (*8*, *9*). For the case of single-line dispatching time optimization, Newell (*10*) analytically showed that to minimize the total passenger waiting time the optimal vehicle dispatching rate varies with time approximately as the square root of the passenger arrival rate. Hurdle (*11*, *12*) extended the work of Newell (*10*) by considering vehicles returning to a dispatching terminal. By using a continuous approximation modeling approach, an optimization model with the objective of minimizing the total passenger waiting time and vehicle operation cost was developed, and the optimal vehicle dispatching rate for different time periods was derived analytically (*11*, *12*). Salzborn (*13*) developed a continuous approximation-based optimization model to optimize the vehicle dispatching times of a bus line to minimize the total passenger waiting time while complying with a fleet size constraint. Stern and Ceder (*14*) developed an integer programming model to optimize the dispatching times with the objective of minimizing both the total passenger waiting time and fleet size. Their model can be solved by using commercial optimization solvers, such as CPLEX. Ceder et al. (*15*) further developed a set of heuristic procedures to generate single-line timetables with either even vehicle headways or even passenger loads in vehicles. In high-frequency services, the objective function of the vehicle dispatching optimization problem is scalar and usually aims to minimize the headway deviation between bus trips of the same service line so as to reduce bus bunching (*16 [Bibr bibr17-03611981221114119]*–*18*). Minimizing this objective function will result in more regular bus operations with reduced average passenger waiting time at bus stops. In the current pandemic, however, additional constraints—such as maintaining a certain level of physical distancing among passengers—become important (*7*, *9*).

The other category of bus dispatching time optimization is in the case of multiple lines, that is, network-wide bus dispatching time optimization. In this case, there are mainly two groups of studies. The first group of studies adopts an equilibrium passenger assignment approach to set the optimal dispatching frequency for each line. This approach takes into account passenger route/trip choice behavior and formulates the optimal dispatching frequency problem as a subproblem of a public transport network design problem. The problem is usually formulated as a bi-level programming model with the upper level objective of minimizing a total system cost and the lower level as an equilibrium passenger assignment problem (*19 [Bibr bibr20-03611981221114119] [Bibr bibr21-03611981221114119]*–*22*). The second group of studies considers coordinating vehicle arrival and departure times at transfer stations when optimizing vehicle dispatching times. The main objective of dispatching time coordination is to minimize the network-wide total passenger transfer waiting time so as to develop seamless transport services. In this case, single-objective or multi-objective integer programming optimization models are developed to optimize the departure and arrival times of vehicles from either a terminal station or intermediate stations (*23 [Bibr bibr24-03611981221114119]*–*25*). Except for these two groups of studies, there are also some other studies considering different objectives and constraints in network-wide bus dispatching time optimization. For example, Furth and Wilson (*26*) developed a mathematical programming model to maximize the net social benefits when optimizing the bus dispatching times for a network of lines.

Nowadays, many public transport service providers are strictly regulated and are instructed to maintain a minimum level of physical distancing inside the vehicles. This has resulted in refusing passenger boarding and skipping stops when the buses reach their pandemic-imposed capacity limit (*2*, *3*, *27*). To mitigate this phenomenon, this study proposes a periodic adjustment optimization model of the dispatching times of planned bus trips that takes into consideration the passenger demand and travel time variations within a rolling horizon optimization framework. A summary and comparisons of previous most relevant studies and our study on single-line bus dispatching optimization are presented in Table 1.

**Table 1.** Comparisons of Relevant Studies on Single-Line Bus Dispatching Optimization

Authors	Objectives	Constraints	Model characteristics	Solution method	Problem description
Newell (*10*)	Minimum passenger waiting time	Fleet size, number of dispatched vehicles	Continuous approximation model	Basic calculus, Lagrange multiplier	Single line without considering returning vehicles
Hurdle (*11*, *12*)	Minimum passenger waiting time, operation cost	Vehicle capacity	Continuous approximation model	Basic calculus, graphical optimization	Single line considering returning vehicles
Stern and Ceder (*14*)	Minimum passenger waiting time, fleet size	Number of dispatched vehicles, service frequency	Integer programming model	Heuristic	Numerical example without considering passenger demand
Ceder et al. (*15*)	Approaching even headway and even passenger loads	Vehicle capacity, headway	Heuristic procedures	Heuristic	Single line with different bus sizes
Berrebi et al. (*17*)	Minimum passenger waiting time	Headway	Stochastic decision process	Backward induction	A loop-shaped route with real-time information
Gkiotsalitis (*9*)	Minimum headway deviation	Headway, slack time	Quadratic programming model	Iterative algorithm using gradient approximations	A high-frequency, circular bus line
This paper	Minimum fleet size, in-vehicle crowding level	Headway, COVID-19 imposed vehicle capacity constraint	Mixed-integer nonlinear programming model	Model liberalization, branch-and-cut (globally optimal solution)	Rescheduling of dispatching times—single line, considering COVID-19 capacity

## Contribution and Organization

The contribution of this paper is threefold. First, we develop a mixed-integer nonlinear programming model to optimize the bus dispatching times of a single bus line considering the COVID-19 imposed vehicle capacity limit and we further reformulate it into a mixed-integer linear program. The optimization model is formulated within a rolling horizon optimization framework to mitigate the uncertainties in vehicle running times and passenger demand. Second, an iterative algorithm is developed to solve the vehicle scheduling and dispatching time rescheduling problem when the number of planned trips is not sufficient to meet the social distancing requirements. At each iteration, a mixed-integer linear program is solved with branch-and-cut. Third, a real-world case study of a bus line in the Twente region in the Netherlands is conducted to demonstrate the effectiveness of the model developed in this study.

The rest of the paper is organized as follows. The next section provides a formal description of the problem studied and its mathematical formulations. This is followed by a presentation of the case study and a discussion of the results. The final section concludes the paper and proposes promising future research directions.

## Problem Description and Model Formulation

### Problem Description

We consider a bus line with ordered stops S={1,2,…,s,…,z} . In the periodic dispatching time control, the dispatching times of several trips that belong to a specific bus line can be updated every time when a new vehicle of the line is about to be dispatched or within a fixed time period (i.e., 1 h). When a trip is about to be dispatched, we consider a fixed time period that includes $N_{m}=\{1,2,\ldots ,m\}$ future trips of the service line and we reschedule the dispatching times of these trips. Note that the members of set $N_{m}$ can change every time when a new vehicle is about to be dispatched (*28*).

Let column vector $\delta ={\left[\delta_{1},\delta_{2},\ldots ,\delta_{m}\right]}^{T}$ with $\delta_{1}\leq \;\delta_{2}\leq \ldots \leq \;\delta_{m}$ be the originally planned dispatching times of trips 1, 2, …, *m*. The originally planned dispatching times δ will be modified by replacing them with the rescheduled dispatching times $x_{1},\;x_{2},\;\ldots ,\;x_{m}$ , such that the in-vehicle passenger crowding levels remain below the pandemic-imposed capacity limit. The rescheduled dispatching times are variables and they are represented by the m -valued vector $x={\left[x_{1},x_{2},\ldots ,x_{m}\right]}^{T}$ where $x\in R_{+}^{m}$ .

Other parameters in our problem include the expected travel time $t_{j,s}$ of trip *j* from stop *s*− 1 to stop *s* and the expected passenger arrival rate at stop *s* for passengers of trip j who are willing to alight at stop *y* during the examined time period, $b_{s,y}$ . These can be represented by an m×(z−1) matrix $T=\{t_{j,s}\}$ and an z×z×m matrix $B=\{b_{s,y}^{j}\}$ . Because travel and passenger arrival rates cannot be negative, $T\in R_{+}^{m\times (z-1)}$ and $B\in R_{+}^{z\times z\times m}$ .

The main assumptions of this work are:

1. The arrivals of passengers at stops are random because for high-frequency bus lines passengers cannot coordinate their arrival time with the arrival time of the bus.
2. The incremental increase of dwell times arising from headway increases is constant (*18*).
3. We do not have a significant number of additional passenger arrivals during the short period that a bus is dwelling at a stop (*29*).

Before proceeding with the formulation of the optimization model, we introduce the nomenclature in Table 2.

**Table 2.** Nomenclature

Sets	
$N_{m}=\{1,2,\ldots ,m\}$	Rescheduled bus trips
S={1,2,…,s,…,z}	Ordered stops of the bus line
Parameters	
$\delta ={\left[\delta_{1},\delta_{2},\ldots ,\delta_{m}\right]}^{T}$	Originally planned dispatching times of trips in $N_{m}$
$T=\{t_{j,s}\}$	Estimated inter-station travel times
$B=\{b_{s,y}^{j}\}$	Expected passenger arrival rate for travelers from stop s to y that will use trip j
c	Pandemic-imposed capacity of each bus
$h_{\min}$	Minimum allowed inter-arrival headway of two consecutive buses at any stop
$r={\left[r_{1},r_{2},\ldots ,r_{s},\;\ldots ,r_{z}\right]}^{T}$	Weight factor that translates the inter-arrival headways at stops to dwell times
τ	Required layover time of a vehicle before starting a new trip
Variables	
$a_{j,s}$	Arrival time of trip j at stop s
$h_{j,s}$	Inter-arrival headway between trips j and j−1 at stop s
$k_{j,s}$	Dwell time of trip j at stop s
$\gamma_{j,s}$	In-vehicle passenger load of trip j when leaving stop s
$e_{i,j}$	Indicator variable of whether trip j can be operated immediately after trip i by using the same vehicle
Decision variables	
$x={\left[x_{1},x_{2},\ldots ,x_{m}\right]}^{T}$	Rescheduled dispatching times
$Y=\{y_{\mathrm{ij}}\}$	0–1 variables where $y_{\mathrm{ij}}=1$ if trips i and j are operated consecutively by the same vehicle, and $y_{\mathrm{ij}}=0$ if not

### Model Formulation

Let us consider a bus trip *j* from the set $N_{m}$ . This bus trip will arrive at each stop *s* of the service line with stops *S* = {1,2, …, *s*, …, z} at time:

(1) $$a_{j,s}\;:=\;a_{j,s-1}\;+\;t_{j,s}\;+\;k_{j,s-1}\;\forall j\in N_{m},s\;\in \;S\;\backslash \;\{1,2\}$$

where $t_{j,s}$ is the expected travel time of trip *j* from stop *s*− 1 to stop *s* and $k_{j,s-1}$ is the dwell time of trip *j* at stop *s*− 1. A special case is the second stop of the service line, where the arrival time is defined according to the boundary condition:

(2) $$a_{j,2}\;:=\;x_{j}\;+\;t_{j,2}\;\forall j\in N_{m}$$

Note that this boundary condition links the arrival time at the second bus stop with the modified dispatching times of the bus trip, $x_{j}$ . Clearly, this will affect all other arrival times at stops *S* = {3, …, *s*, …, z} because Equation 1 is a recurrence relation that uses Equation 2 as its initial value. We note that the arrival times are variables that can be represented by a matrix $A=\{a_{j,s}\}$ with $A\in R_{+}^{m\times z}$ . In addition, the dwell times are variables that can be represented by matrix $K=\{k_{j,s}\}$ with $\;K\in R_{+\;}^{m\times (z-1)}$ .

To avoid bus bunching we need to ensure that there is a minimum headway, $h_{\min}$ , between two successive trips j,j+1 when arriving at the same stop s . This is achieved by imposing the inequality constraints:

(3) $$a_{j,s}+h_{\min}\leq \;a_{j+1,s}\;\forall j\in N_{m}\backslash \{m\},\;s\;\in \;S$$

where $h_{\min}>0$ is a constant. The inter-arrival time headway between two consecutive bus trips at a stop *s* is also computed as:

(4) $$h_{j,s}\;:=\;a_{j,s}\;-\;a_{j-1,s}\;\forall j\;\in \;N_{m}\;\backslash \;\left\{1\right\},s\in S$$

and it is a variable $H=\left\{h_{i,j}\right\}.$

In addition, as described in assumption 2, the dwell time at stops for performing boardings/alightings is proportional to the time headway between two successive bus trips, $k_{j,s}\propto h_{j,s}$ . That is, the elements of matrix $K=\{k_{j,s}\}$ are variables that take values from:

(5) $$k_{j,s}\;:=\;r_{s}\;h_{j,s}\;\forall j\;\in \;N_{m}\;\backslash \;\left\{1\right\},s\in S\backslash \{z\}$$

where $r_{s}$ is a parameter with a positive value indicating the linear relationship between the dwell time and the time headway (the longer the time headway, the higher the dwell time because there are more passenger arrivals at the bus stop). This parameter can change its value from stop to stop and it is represented by a vector $r={\left[r_{1},r_{2},\ldots ,r_{s},\;\ldots ,r_{z}\right]}^{T}$ . For instance, Daganzo (*18*) reported a $r_{s}\approx 0.01$ .

In addition, the in-vehicle passenger load of a trip *j* on departing from the first stop *s* = 1 is a variable:

(6) $$\gamma_{j,1}:=\sum_{y\in S}b_{1,y}^{j}h_{j,1}\;\forall j\in N_{m}\backslash \{1\}$$

At any other stop *s* > 1 the in-vehicle passenger load becomes:

(7) $$\gamma_{j,s}:=\gamma_{j,s-1}+\sum_{y\in S|y>s}b_{s,y}^{j}h_{j,s}-\;\sum_{y\in S|y<s}b_{y,s}h_{j,y}\;\forall j\in N_{m}\backslash \{1\},s\in S\backslash \{1\}$$

where the added sum indicates the boarding passengers at stop *s* that will alight at any other stop *y* > *s* and the subtracted sum indicates the boarded passengers at previous stops *y* < *s* who alight at stop *s*. Note that Equation 7 ensures the conservation of passenger flow. The in-vehicle passenger load on departing from each stop can be represented by a matrix $\Gamma =\{\gamma_{j,s}\}$ where $\Gamma \in R_{+}^{m\times z}$ .

Let *c* be the pandemic-imposed capacity for the vehicles of the service line (parameter). Then, we seek a rescheduling solution x that does not result in in-vehicle passenger loads beyond the pandemic-imposed capacity limit after departing from any stop *s*:

(8) $$\gamma_{j,s}\leq c\;\forall j\in N_{m},s\in S$$

Enforcing constraint (8) is implicitly the main aim of this study because it will ensure that we do not have more in-vehicle passengers than the pandemic-imposed capacity limit. Achieving this, however, might result in the requirement of more vehicles to perform the rescheduled service. To rectify this, we seek to maximize the number of trips performed by each vehicle while meeting the new schedule. We thus solve the Single-Depot Vehicle Scheduling Problem (SD-VSP) that strives to deploy the minimum number of vehicles that are parked at a single depot to perform the rescheduled plan of the $N_{m}=\{1,2,\ldots ,m\}$ trips.

Based on the new rescheduled dispatching times, x , we first introduce indicator variables $E=\{e_{\mathrm{ij}}\}$ where $e_{\mathrm{ij}}=1$ if trip j can be operated after trip i by using the same vehicle and $e_{\mathrm{ij}}=-M$ , where −M is a very large negative number if it cannot. Trip j can be operated after trip i if the starting time of trip j , $x_{j}$ , is greater than the time trip i arrived at the last stop z plus some layover time τ . That is,

(9a) $$e_{i,j}:=\{\begin{array}{c} 1\;\;\mathrm{if}\;x_{j}\geq a_{i,z}+\tau \\ -M\;\;\mathrm{otherwise}. \end{array}\;\;\forall i\in N_{m},j\in N_{m}$$

Equation 9a is a conditional expression. If trip j can be operated after trip i by using the same vehicle, $x_{j}\geq a_{i,z}+\tau$ , then $e_{\mathrm{ij}}=1$ . If not, $x_{j}<a_{i,z}+\tau$ which results in $e_{i,j}$ receiving a very large negative value $e_{i,j}=-M$ .

To linearize it, we first introduce binary variables $D=\{d_{\mathrm{ij}}\}$ where $d_{\mathrm{ij}}=1$ if $x_{j}-a_{i,z}-\tau \geq 0$ and $d_{\mathrm{ij}}=0$ if $x_{j}-a_{i,z}-\tau <0$ . This can be imposed by the following inequality constraints.

(9b) $$\begin{array}{c} x_{j}-a_{i,z}-\tau <Md_{\mathrm{ij}}\;\forall i\in N_{m},j\in N_{m}\\ x_{j}-a_{i,z}-\tau \geq M\left(d_{\mathrm{ij}}-1\right)\;\forall i\in N_{m},j\in N_{m}\\ d_{\mathrm{ij}}\in \{0,1\} \end{array}$$

Using the values of $d_{\mathrm{ij}}$ , we can now replace the conditional expression of Equation 9a with the following constraints:

(9c) $$e_{i,j}:=1-(M-1)(1-d_{\mathrm{ij}})\;\forall i\in N_{m},j\in N_{m}$$

The equisatisfiability of Equations 9a to 9c that allows us to replace the conditional expression (9a) by the set of linear equality and inequality constraints in (9b) to (9c) is proved in the following Lemma.

**Lemma 1.** Constraints

$$e_{i,j}=\{\begin{array}{c} 1\;\;\mathrm{if}\;x_{j}\geq a_{i,z}+\tau \;\\ -M\;\;\mathrm{otherwise}. \end{array}\;\forall i\in N_{m},j\in N_{m}$$

and

$$\begin{array}{c} x_{j}-a_{i,z}-\tau <Md_{\mathrm{ij}}\;\forall i\in N_{m},j\in N_{m}\\ x_{j}-a_{i,z}-\tau \geq M\left(d_{\mathrm{ij}}-1\right)\;\forall i\in N_{m},j\in N_{m} \end{array}$$

$$\begin{array}{c} d_{\mathrm{ij}}\in \{0,1\}\\ e_{i,j}=1-(M-1)(1-d_{\mathrm{ij}})\;\forall i\in N_{m},j\in N_{m} \end{array}$$

are equisatisfiable.

Proof : It is sufficient to prove that Equations 9b and 9c return $e_{i,j}=1$ when $x_{j}>a_{i,z}+\tau$ or $x_{j}=a_{i,z}+\tau$ , and $e_{i,j}=0$ when $x_{j}<a_{i,z}+\tau$ . We present these three cases:

Case I, $x_{j}>a_{i,z}+\tau$ : in this case, $x_{j}-a_{i,z}-\tau >0$ . Thus, $x_{j}-a_{i,z}-\tau \geq M\left(d_{\mathrm{ij}}-1\right)$ is satisfied for both $d_{\mathrm{ij}}=0$ or $d_{\mathrm{ij}}=1$ , but constraint $x_{j}-a_{i,z}-\tau <Md_{\mathrm{ij}}$ is satisfied if, and only if, $d_{\mathrm{ij}}=1$ . Thus, $d_{\mathrm{ij}}=1$ to satisfy (9b). To satisfy (9c) for $d_{\mathrm{ij}}=1$ we have $e_{i,j}:=1-\left(M-1\right)\left(1-1\right)=1$ . This proves that $e_{i,j}=1$ for $x_{j}-a_{i,z}-\tau >0$ .
Case II, $x_{j}=a_{i,z}+\tau$ : in this case, $x_{j}-a_{i,z}-\tau =0$ . Thus, $x_{j}-a_{i,z}-\tau \geq M\left(d_{\mathrm{ij}}-1\right)$ is satisfied for both $d_{\mathrm{ij}}=0$ or $d_{\mathrm{ij}}=1$ , but constraint $x_{j}-a_{i,z}-\tau <Md_{\mathrm{ij}}$ is satisfied if, and only if, $d_{\mathrm{ij}}=1$ . Thus, $d_{\mathrm{ij}}=1$ to satisfy (9b). To satisfy (9c) for $d_{\mathrm{ij}}=1$ , $e_{i,j}=1$ .
Case III, $x_{j}<a_{i,z}+\tau$ : in this case, $x_{j}-a_{i,z}-\tau <0$ . Thus, $x_{j}-a_{i,z}-\tau <Md_{\mathrm{ij}}$ is satisfied for both $d_{\mathrm{ij}}=0$ or $d_{\mathrm{ij}}=1$ , but constraint $x_{j}-a_{i,z}-\tau \geq M\left(d_{\mathrm{ij}}-1\right)$ is satisfied if, and only if, $d_{\mathrm{ij}}=0$ . Thus, $d_{\mathrm{ij}}=0$ to satisfy (9c). To satisfy (9c) for $d_{\mathrm{ij}}=0$ we have $e_{i,j}:=1-\left(M-1\right)\left(1-0\right)=1-M-1=-M$ . This proves that $e_{i,j}=-M$ for $x_{j}-a_{i,z}-\tau >0$ and completes our proof.

Lemma 1 proved that the conditional expression Equation 9a can be replaced by the linear constraints (9b) to (9c) that will be referred to as Equation 9 at the remainder of this paper.

Let us also introduce binary variables $Y=\{y_{\mathrm{ij}}\}$ where $y_{\mathrm{ij}}=1$ if trips i and j are operated by the same vehicle and $y_{\mathrm{ij}}=0$ if they are not. Because if a vehicle serves a trip i it can only serve at most one trip j immediately after serving trip i we have:

(10) $$\sum_{j\in N_{m}}y_{\mathrm{ij}}\leq 1\;\forall i\in N_{m}$$

In addition, if a vehicle serves trip j it could have served before trip j at most one trip:

(11) $$\sum_{i\in N_{m}}y_{\mathrm{ij}}\leq 1\;\forall j\in N_{m}$$

Equations 10 and 11 ensure that if two trips i and j are served subsequently by the same vehicle, then there is no other intermediate trip between them. Using this formulation, we formalize the mathematical program:

$$\begin{array}{c} \max\;\sum_{i\in N_{m}}\sum_{j\in N_{m}}y_{\mathrm{ij}}e_{\mathrm{ij}}\\ \mathrm{subject}\;\mathrm{to}:\;\mathrm{Eqs}.\left(1\right)-\left(11\right) \end{array}$$

$$x\in R^{m},Y\in \{0,1{\}}^{m\times m}$$

The objective function of the mathematical program is nonlinear. Note that because the problem is a maximization problem, when two subsequent trips i,j cannot be served by the same vehicle and $e_{\mathrm{ij}}=-M$ , then $y_{\mathrm{ij}}$ will be forced to be equal to 0 to not allow a steep reduction in the objective function. This is why $e_{\mathrm{ij}}$ was set equal to −M in the first Equation 9a.

Constraints (1) to (11) are linear. In addition, the mathematical program has both continuous and discrete variables, and it is a mixed-integer nonlinear programming problem (MINLP). The continuous relaxation of the bus rescheduling problem expressed in our MINLP does not have a concave objective function that would have allowed us to compute a globally optimal solution. This is formalized in the following theorem.

**Theorem 1.** The continuous relaxation of the MINLP does not have a concave objective function and a local maximizer of the problem is not necessarily a global maximizer.

Proof: Considering the continuous relaxation of the MINLP, the feasible region of the problem is a convex set because the finite number of linear inequalities forms a polyhedron. However, the objective function is not concave. Let $g_{i,j}=y_{\mathrm{ij}}e_{\mathrm{ij}}$ . Then, the Hessian matrix of $g_{i,j}$ is:

$$H\left(g_{i,j}\right)=\;\left[\begin{array}{cc} \frac{d^{2}g_{i,j}}{{\mathrm{dy}}_{\mathrm{ij}}^{2}} & \frac{d^{2}g_{i,j}}{dy_{\mathrm{ij}}de_{\mathrm{ij}}}\\ \frac{d^{2}g_{i,j}}{de_{\mathrm{ij}}dy_{\mathrm{ij}}} & \frac{d^{2}g_{i,j}}{{\mathrm{de}}_{\mathrm{ij}}^{2}} \end{array}\right]\;=\;\left[\begin{array}{cc} 0\; & 1\\ 1 & 0 \end{array}\right]$$

with eigenvalues $\lambda ={\left[-1,1\right]}^{T}.$ Because the eigenvalues are both positive and negative, the Hessian matrix is indefinite and the objective function is not convex or concave. This completes the proof.

### Reformulation to a Mixed-Integer Linear Program

Our objective function is both nonlinear and not convex and this does not allow us to guarantee the global optimality of the solutions of our MINLP’s continuous relaxations. To rectify this, we replace each product $y_{\mathrm{ij}}e_{\mathrm{ij}}$ in our objective function by a separable function $m_{\mathrm{ij}}^{2}-l_{\mathrm{ij}}^{2}$ , where:

(12) $$m_{\mathrm{ij}}=\frac{1}{2}\left(y_{\mathrm{ij}}+e_{\mathrm{ij}}\right)\;\forall i\in N_{m},j\in N_{m}$$

(13) $$\begin{array}{c} l_{\mathrm{ij}}=\frac{1}{2}\left(y_{\mathrm{ij}}-e_{\mathrm{ij}}\right)\;\forall i\in N_{m},j\in N_{m}\\ m_{\mathrm{ij}}\in R,\;l_{\mathrm{ij}}\in R\;\forall i\in N_{m},j\in N_{m} \end{array}$$

Observe that $m_{\mathrm{ij}}^{2}-l_{\mathrm{ij}}^{2}=y_{\mathrm{ij}}e_{\mathrm{ij}}$ because $m_{\mathrm{ij}}^{2}-l_{\mathrm{ij}}^{2}=\frac{1}{4}(y_{\mathrm{ij}}^{2}+2y_{\mathrm{ij}}e_{\mathrm{ij}}+e_{\mathrm{ij}}^{2})-\frac{1}{4}(y_{\mathrm{ij}}^{2}-2y_{\mathrm{ij}}e_{\mathrm{ij}}+e_{\mathrm{ij}}^{2})=y_{\mathrm{ij}}e_{\mathrm{ij}}$ . Now we have a separable function $m_{\mathrm{ij}}^{2}-l_{\mathrm{ij}}^{2}$ instead of the nonseparable $y_{\mathrm{ij}}e_{\mathrm{ij}}$ . Notice that $m_{\mathrm{ij}}^{2}-l_{\mathrm{ij}}^{2}$ is still nonlinear. $m_{\mathrm{ij}}^{2}$ and $l_{\mathrm{ij}}^{2}$ are both quadratic functions $g\left(m_{\mathrm{ij}}\right)=m_{\mathrm{ij}}^{2}$ and $\tilde{g}\left(l_{\mathrm{ij}}\right)=l_{\mathrm{ij}}^{2}$ . Both can be approximated by piecewise linear functions. Let us consider $\left(m_{\mathrm{ij}}\right)=m_{\mathrm{ij}}^{2}$ . If we compute the values $m_{a,\mathrm{ij}}^{2},m_{b,\mathrm{ij}}^{2},\ldots ,m_{n,\mathrm{ij}}^{2}$ at the breakpoints of the piecewise linear function, $m_{a,\mathrm{ij}},m_{b,\mathrm{ij}},\ldots ,m_{n,\mathrm{ij}}$ , then $g\left(m_{\mathrm{ij}}\right)=m_{\mathrm{ij}}^{2}$ can be approximated as follows:

(14a) $$g\left(m_{\mathrm{ij}}\right)\approx \mu_{a,\mathrm{ij}}m_{a,\mathrm{ij}}^{2}+\mu_{b,\mathrm{ij}}m_{b,\mathrm{ij}}^{2}\ldots +\mu_{n}m_{n,\mathrm{ij}}^{2}\;\forall i\in N_{m},j\in N_{m}$$

(14b) $$m_{\mathrm{ij}}=\mu_{a,\mathrm{ij}}m_{a,\mathrm{ij}}\;+\mu_{b,\mathrm{ij}}m_{b,\mathrm{ij}}+\ldots +\mu_{n,\mathrm{ij}}m_{n,\mathrm{ij}}\;\forall i\in N_{m},j\in N_{m}$$

(14c) $$\sum_{y=a}^{n}\mu_{y,\mathrm{ij}}=1\;\forall i\in N_{m},j\in N_{m}$$

(14d) $$\mu_{y,\mathrm{ij}}\geq 0\;\forall y\in \left\{a,\ldots ,n\right\},i\in N_{m},j\in N_{m}$$

(14e) $$\mathrm{SOS}2(\mu_{a,\mathrm{ij}},\mu_{b,\mathrm{ij}},\ldots ,\mu_{n,\mathrm{ij}})\;\forall i\in N_{m},j\in N_{m}$$

where special ordered sets of type 2 (SOS2) ( $\mu_{a,\mathrm{ij}},\mu_{b,\mathrm{ij}},\ldots ,\mu_{n,\mathrm{ij}})$ are the constraints for SOS2 which force variables $\mu_{a,\mathrm{ij}},\mu_{b,\mathrm{ij}},\ldots ,\mu_{n,\mathrm{ij}}$ to receive values such that at most two of them can be non-zero, and if two of them are non-zero, they must be adjacent to each other based on their predefined ordering (*30*). Similar constraints can be applied for approximating $\tilde{g}\left(l_{\mathrm{ij}}\right)=l_{\mathrm{ij}}^{2}$ with a piecewise linear function:

(15a) $$g\left(l_{\mathrm{ij}}\right)\approx \lambda_{a,\mathrm{ij}}l_{a,\mathrm{ij}}^{2}+\lambda_{b,\mathrm{ij}}l_{b,\mathrm{ij}}^{2}\ldots +\lambda_{n}l_{n,\mathrm{ij}}^{2}\;\forall i\in N_{m},j\in N_{m}$$

(15b) $$l_{\mathrm{ij}}=\lambda_{a,\mathrm{ij}}l_{a,\mathrm{ij}}\;+\lambda_{b,\mathrm{ij}}l_{b,\mathrm{ij}}+\ldots +\lambda_{n,\mathrm{ij}}l_{n,\mathrm{ij}}\;\forall i\in N_{m},j\in N_{m}$$

(15c) $$\sum_{y=a}^{n}\lambda_{y,\mathrm{ij}}=1\;\forall i\in N_{m},j\in N_{m}$$

(15d) $$\lambda_{y,\mathrm{ij}}\geq 0\;\forall y\in \left\{a,\ldots ,n\right\},i\in N_{m},j\in N_{m}$$

(15e) $$\mathrm{SOS}2(\lambda_{a,\mathrm{ij}},\lambda_{b,\mathrm{ij}},\ldots ,\lambda_{n,\mathrm{ij}})\;\forall i\in N_{m},j\in N_{m}$$

Thus, our MINLP is reformulated to the mixed-integer linear program (MILP) as:

$$\begin{array}{c} \max\;\sum_{i\in N_{m}}\sum_{j\in N_{m}}\left(g\left(m_{\mathrm{ij}}\right)-\tilde{g}(l_{\mathrm{ij}})\right)\\ \mathrm{subject}\;\mathrm{to}:\;\mathrm{Eqs}.\left(1\right)-\left(15\right)\\ x\in R^{m},Y\in \{0,1{\}}^{m\times m} \end{array}$$

This new mathematical program is easier to solve because its objective function is separable and piecewise linear, resulting in a MILP.

### The Decision Problem of Adding More Trips when We Cannot Guarantee Physical Distancing

When solving the MILP to determine the rescheduled dispatching times x and the vehicle schedules Y , it is important to note that the MILP has a feasible solution if, and only if, the problem instance has enough trips $N_{m}=\{1,2,\ldots ,m\}$ to satisfy the physical distancing constraint of Equation 8 when planning the dispatching times in an optimal way. This is a very important remark because a problem instance can result in one of the following cases:

1. The physical distancing is already satisfied by the originally planned schedule.
2. The physical distancing is not satisfied by the originally planned schedule and we need to perform rescheduling by changing the dispatching times of trips.
3. The physical distancing is not satisfied even after performing an optimal rescheduling because the number of trips is not enough.

In the third case, our MILP does not have a feasible solution. It is important to note that if a feasible solution does not exist because the physical distancing constraint in Equation 8 cannot be satisfied despite the values of x , Y , then one should add one more trip in the examined time period and solve again the MILP. If there is still no feasible solution, more trips should be added in the examined time period until finding a feasible solution. This can be expressed as a decision problem with a “yes” or “no” answer as follows:

Decision Problem: “For a given bus line with $N_{m}=\{1,2,\ldots ,m\}$ planned trips in the next time horizon and passenger arrival rates B , is there an optimal rescheduling option ( x , Y) that can ensure the physical distancing of passengers at all stops?”

The aforementioned decision problem indicates that changing the dispatching times of trips $N_{m}=\{1,2,\ldots ,m\}$ is not always sufficient to ensure that the pandemic-imposed capacity constraint is satisfied. In that case, trips should be added incrementally, and the MILP should be solved repeatedly until the physical distancing requirement is satisfied at all stops. For instance, if in a time horizon of 2 h we have m=12 planned trips and there is no rescheduling solution with 12 trips that can meet the pandemic-imposed capacity limit, then we can add one more trip and solve the MILP again to find the new dispatching times and the number of vehicles required. In practice, this can be performed by Algorithm 1 which increases incrementally the number of trips m in the decision problem until the decision problem has a “yes” answer for some x , Y .

**Table:** 

**Algorithm 1**: Vehicle scheduling and dispatching time rescheduling until meeting the physical distancing requirement.	
1:	Start from the current schedule with trips $N_{m}=\{1,2,\ldots ,\;m\}$
2:	**If** (solving the MILP with m trips returns a feasible solution):
3:	Return new dispatching times and vehicle schedules x,Y and terminate.
4:	**Else:**
5:	**Repeat:**
6:	Add one more trip in this time period { 1,2,…,m+1}
7:	Set m←m+1
8:	Solve the new MILP
9:	**Until:** solving the MILP returns a feasible solution
10:	Return new schedules x ,**Y**.

We note that this decision problem is in NP (it belongs to the Nondeterministic Polynomial time class of problems) because:

• it requires exponential time to solve the MILP since the computation steps increase exponentially with the size of Y ;
• if an oracle provides a solution x , Y to a “yes” instance of the decision problem, we can verify whether the solution x , Y is a feasible solution or not in polynomial time by plugging the values of x , Y in Equations 1 to 11.

## Case Study and Results

### Case Study Problem Description

**Figure 1.**
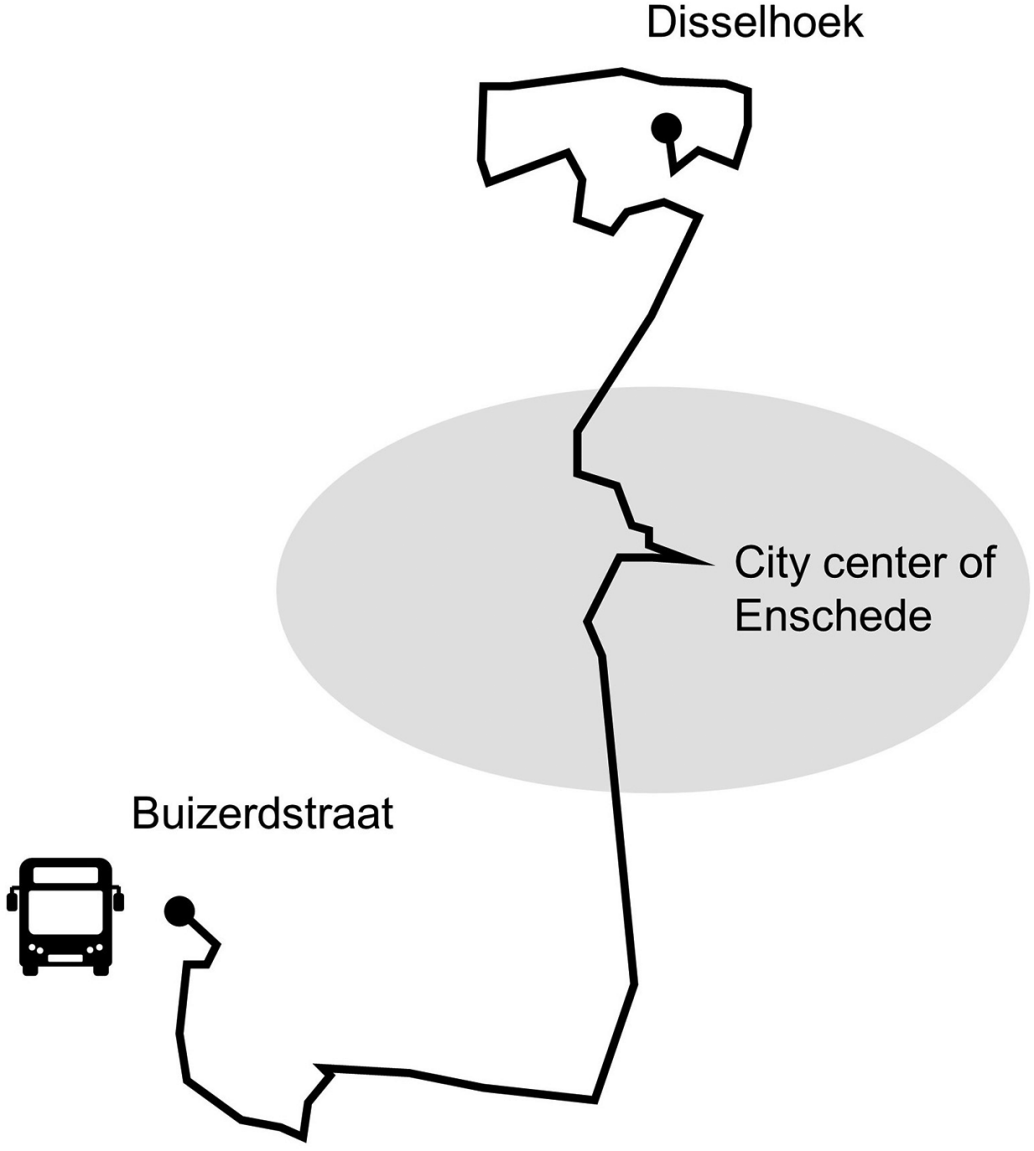
Topology of bus Line 2 that passes through the city center of Enschede.

The proposed model is tested in a simulation of bus Line 2 in the Twente region that connects the southern districts with the northern districts of Enschede (a city of approximately 160,000 inhabitants). In this simulation, we use real passenger demand data from the March 23, 2020. This bus line is selected because it has specific line segments with abnormally high passenger demand levels that exceed the pandemic-imposed capacity (see Figure 1). The line has a total of 40 stops. The line’s length is around 13 km and its average travel time in the morning peak is about 43 min. We consider an average inter-station travel time $t_{\mathrm{js}}$ of 65.3 s. The pandemic-imposed capacity is c=15 passengers. In addition, the dwell time is proportional to the inter-arrival headway of buses at the stops by multiplying that time by r=0.01 as proposed in Daganzo (*18*)—see survey of Gkiotsalitis and Cats (*31*).

In our case study we use passenger demand data from March 23, 2020 which was one of the first days when the pandemic regulations were imposed in the Netherlands. We focus on two time periods:

1. Mild Passenger Demand Time period from 13:00 until 14:00 with six trips planned to be dispatched at $\left(\delta_{1},\delta_{2},\delta_{3},\delta_{4},\delta_{5},\delta_{6}\right)$ =(13:00,13:10,13:20,13:30,13:40,13:50)
2. Peak Passenger Demand Time period from 08:00 until 09:00 with six trips planned to be dispatched at $\left(\delta_{1},\delta_{2},\delta_{3},\delta_{4},\delta_{5},\delta_{6}\right)$ =(08:00,08:10,08:20,08:30,08:40,08:50)

For both time periods we report the in-vehicle passenger load of the buses when the schedule is operated as planned, and when we make dispatching time, vehicle scheduling, and trip changes. This demonstrates the improvement potential of our model in finding solutions that can ensure physical distancing and the potential increase in operational costs in case more trips are needed.

Our Algorithm 1 is implemented in Python 3.7 and our MILP that provides the optimal dispatching times and vehicle schedules is programmed in Python and it is solved by the commercial solver Gurobi 9.0.3 that employs branch-and-cut for solving MILPs. The experiments are run on a general-purpose computer with Intel Core i7-7700HQ CPU @ 2.80 GHz and 16 GB RAM.

### Results Under Mild Passenger Demand

We first reschedule the trip dispatching times and the vehicle schedules under the mild demand scenario. This is achieved by solving our MILP with a branch-and-cut algorithm. Our MILP was solved to optimality, meaning that the available number of six trips suffice to meet the physical distancing constraint when the trips are rescheduled appropriately. The solution of the solver was provided in less than 2 s and it is:

• *y*_16_ = 1and $y_{\mathrm{ij}}=0$ for all other $i\in N_{m},\;j\in \;N_{m}$
• **x**=[0,8,16,27,38,38]^T^, where the values are expressed in minutes after 13:00, that is, 8 refers to 13:08 and 16 to 13:16.

To elaborate more on the computational complexity of our model, even if we consider very frequent service lines with 2-min time headways resulting in 30 trips per hour, the number of binary decision variables remains small (we would have 900 $y_{\mathrm{ij}}$ variables). Even if we consider a large peak period that can last for 3 h, we do not have more than 8,100 binary $y_{\mathrm{ij}}$ variables and the problem is still tractable. Intractability issues may occur if one considers all daily trips on a very busy line with 30 trips per hour. However, this is very rare in practice because it is highly unlikely that a line would be crowded throughout the entire day.

The original and the rescheduled dispatching times are presented in Table 3 together with the vehicle that is assigned to every trip. Note that in both cases we need five vehicles to operate the six trips from 13:00 to 14:00.

**Table 3.** Planned and Rescheduled Dispatching Times from 13:00 to 14:00 when the Passenger Demand is Mild

Trips $N_{m}$	1	2	3	4	5	6
Planned						
Dispatching time $\delta_{i}$	13:00	13:10	13:20	13:30	13:40	13:50
Vehicle that operates the trip	1	2	3	4	5	1
Rescheduled						
Rescheduled dispatching time $x_{i}$	13:00	13:08	13:16	13:27	13:38	13:50
Vehicle that operates the trip	1	2	3	4	5	1

**Figure 2.**
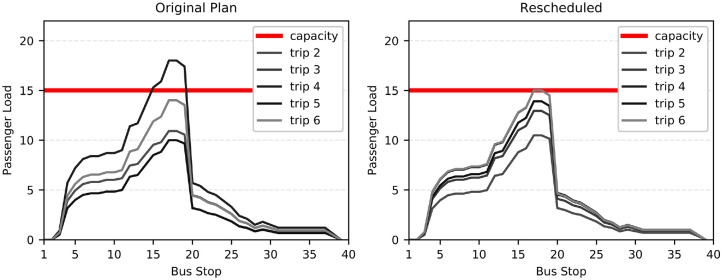
Passenger load at every stop of bus Line 2 from 13:00 to 14:00 on March 23, 2020.

The in-vehicle crowding levels before and after the implementation of the rescheduling model are presented in Figure 2. The originally planned (before) dispatching times resulted in in-vehicle overcrowding in five inter-station links and in an excessive number of three onboard passengers beyond the pandemic-imposed capacity (18 passengers instead of 15). To make this more concrete, we introduce the key performance indicator of “passenger-km” which multiplies the number of passengers beyond the pandemic capacity level by the km traveled beyond that capacity. This results in a value of 3.2 passenger-km when implementing the original plan. When implementing the rescheduled dispatching times, this issue is resolved. As can be seen in Figure 2, the rescheduled dispatching times do not result in in-vehicle crowding levels beyond the 15-passenger limit.

### Results Under Peak Passenger Demand

We now reschedule the trip dispatching times and the vehicle schedules under the peak demand scenario for the time period from 08:00 to 09:00. Given the six planned trips, it was not possible to solve our MILP because a feasible solution did not exist. That is, the following decision problem does not have a “yes” answer:

Decision Problem: “For bus line 2 with $N_{m}=\{1,2,\ldots ,6\}$ planned trips from 08:00 to 09:00 and passenger arrival rates B , is there an optimal rescheduling option ( x , Y) that can ensure the physical distancing of passengers at all stops?”

Because the number of planned trips is not enough to ensure physical distancing, we implement Algorithm 1 by increasing incrementally the number of trips until our MILP can return a feasible solution, which is the optimal one for this number of trips. After increasing the number of trips, it was possible to solve our MILP when considering nine trips from 08:00 to 09:00. The solution of the solver when considering nine trips is:

• *y*_19_ = 1 and $y_{\mathrm{ij}}=0$ for all other $i\in N_{m},\;j\in \;N_{m}$
• **x**=[0,9,14,19,26,32,38,44,50]^T^ where the values are expressed in minutes after 08:00, that is, 9 refers to 08:09.

The original and the rescheduled dispatching times are presented in Table 4 together with the vehicle that is assigned to every trip. Note that when implementing the original plan we need five vehicles to operate the six trips and when operating the rescheduled plan that ensures physical distancing we need eight vehicles to operate nine trips from 08:00 to 09:00.

**Table 4.** Planned and Rescheduled Dispatching Times From 08:00 to 09:00 When the Passenger Demand is in a Peak

Trips $N_{m}$	1	2	3	4	5	6	7	8	9
Planned									
Dispatching time $\delta_{i}$	08:00	08:10	08:20	08:30	08:40	08:50	NA	NA	NA
Vehicle that operates the trip	1	2	3	4	5	1			
Rescheduled									
Rescheduled dispatching time $x_{i}$	08:00	08:09	08:14	08:19	08:26	08:32	08:38	08:44	08:50
Vehicle that operates the trip	1	2	3	4	5	6	7	8	1

*Note*: NA = Not Available.

**Figure 3.**
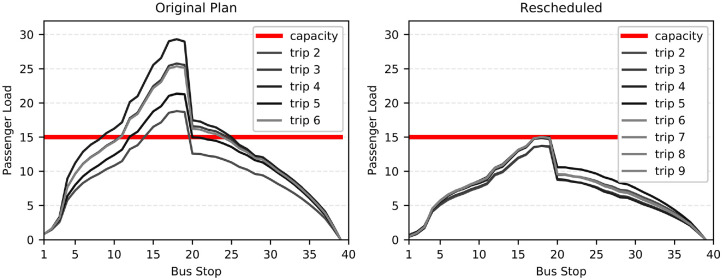
Passenger load at every stop of bus Line 2 from 08:00 to 09:00 on March 23, 2020.

The in-vehicle crowding levels before and after the implementation of the rescheduling model are presented in Figure 3. The originally planned (before) dispatching times resulted in in-vehicle overcrowding in several inter-station links for all trips. To make this more concrete, we consider again the “passenger-km” indicator which multiplies the number of passengers beyond the pandemic capacity level by the km traveled beyond that capacity. This results in a value of 89 passenger-km when implementing the original plan. When implementing the rescheduled dispatching times, this issue is resolved. As can be seen in Figure 3, the rescheduled dispatching times do not result in in-vehicle crowding levels beyond the 15-passenger limit. It is clear that our rescheduling model can always generate vehicle passenger loads that satisfy the COVID-19 imposed vehicle capacity limit. It can be a valuable decision-making tool for supporting the daily vehicle scheduling and rescheduling activities of public transport operators.

### Policy Implications

Social distancing measures can contribute to reducing public transport passengers’ risk of contracting COVID-19. However, they also lead to a reduction in the usage of public transport vehicles. The onboard passengers in a public transport vehicle often exceed the COVID-19 imposed vehicle capacity limit, especially during peak hours. If the originally planned vehicle dispatching times are properly rescheduled based on passenger demand, the excessive crowding can be eliminated. There are cases, however, where a mere change in the dispatching times is not sufficient. This should be considered when making policy-related decisions. For instance, in our case study, we required three more vehicles during the peak hour to offer a service that meets the COVID-19 vehicle capacity. This is a 60% increase compared with the case where the ordinary capacity is considered. If all lines in the network have a passenger demand peak at the same hour of the day, such an increase will most probably consume the available resources of the transit operator and will require the purchase of new vehicles to meet the demand peak.

Our study underlines the nontrivial decisions that should be made by policymakers and quantifies the potential extra costs of adopting a COVID-19 capacity limit. Our formulation can be used to test the effects of different COVID-19 capacity limit values to the extra number of required vehicles to establish a satisfactory trade-off between the risk of virus contraction and operating costs.

## Conclusion

To prevent the spreading of COVID-19, different physical distancing strategies are adopted by public transport service providers. Although different physical distancing regulations are recommended to service providers by the government, it is a very complex task to change the originally planned services to meet the physical distancing requirements under different demand scenarios. This study tried to propose an optimization model in this direction that uses short-term passenger demand information with regard to the expected passenger arrival rates in the hours ahead to modify the dispatching times of the trips and the vehicle schedules. This study introduced a mixed-integer nonlinear programming problem formulation to perform this task which was later reformulated to a mixed-integer linear programming problem. In addition, this study formulated the decision problem of determining whether the planned number of trips suffices to meet the physical distancing requirement or there is a need to increase the number of trips by using additional vehicles.

The proposed approach was tested on a bus line in the Netherlands using data from the early stages of the COVID-19 pandemic when the physical distancing restrictions were stricter. To explore the effect of this method under different demand scenarios, we explored both mild and peak demand problem instances. Under mild demand conditions, a simple rescheduling of the dispatching times of trips was enough to meet the physical distancing requirements and to avoid the 3.2 passenger-km traveled with an in-vehicle load beyond the physical distancing level. Under peak demand conditions, however, the situation was different. To meet the physical distancing requirement, we needed to deploy three more vehicles and increase the number of trips from six to nine. Only then we were able to avoid the 89 passenger-km traveled with an in-vehicle load beyond the physical distancing level that was observed when implementing the original plan. This information could be very useful to service providers who want to balance the operational cost increases with the physical distancing requirements.

In future research, our approach can be further expanded to perform real-time control measures, such as bus holding or stop-skipping, that can reduce the number of required vehicles when trying to maintain physical distancing. In addition, transfers between bus and train lines can be considered to avoid disproportionate passenger demand increases inside trains and at train stations.
